# Prognostic value of soluble TNF receptors in Chagas cardiomyopathy: observational study

**DOI:** 10.1590/0074-02760240107

**Published:** 2025-04-07

**Authors:** Matheus Ribeiro Ávila, Daniel Menezes de Souza, Lucas Fróis Fernandes de Oliveira, Pedro Henrique Scheidt Figueiredo, Liliany Mara Carvalho Silva, Whesley Tanor Silva, Mauro Felippe Felix Mediano, Leonardo Augusto da Costa Teixeira, Luciano Fonseca Lemos de Oliveira, Marcus Alessandro de Alcantara, Sanny Cristina de Castro Faria, Arthur Nascimento Arrieiro, Vanessa Amaral Mendonça, Ana Cristina Rodrigues Lacerda, Henrique Silveira Costa

**Affiliations:** 1 Universidade Federal dos Vales do Jequitinhonha e Mucuri, Programa de Pós-Graduação em Reabilitação e Desempenho Funcional, Diamantina, MG, Brasil; 2 Universidade Federal de Minas Gerais, Programa de Pós-Graduação em Infectologia e Medicina Tropical, Belo Horizonte, MG, Brasil; 3 Universidade Federal dos Vales do Jequitinhonha e Mucuri, Programa de Pós-Graduação em Ciências da Saúde, Diamantina, MG, Brasil; 4 Fundação Oswaldo Cruz-Fiocruz, Instituto Nacional de Infectologia Evandro Chagas, Rio de Janeiro, RJ, Brasil; 5 Universidade Federal de Minas Gerais, Escola de Educação Física, Fisioterapia e Terapia Ocupacional, Programa de Pós-Graduação em Ciências da Reabilitação, Belo Horizonte, MG, Brasil

**Keywords:** Chagas disease, Chagas cardiomyopathy, biomarkers, prognosis

## Abstract

**BACKGROUND:**

Chronic Chagas cardiomyopathy (CCC) is the most severe clinical form of the Chagas disease. There is a strong correlation between soluble tumor necrosis factor receptors (sTNFR1 and sTNFR2) and cardiac and functional parameters in CCC, but their prognostic value remains unknown.

**OBJECTIVE:**

To verify the prognostic value of sTNFR1 and sTNFR2 in CCC.

**METHODS:**

A longitudinal study was conducted. Sixty-nine patients with CCC (53.70 ± 9.66 years, NYHA I-II) were submitted to blood collection and echocardiography, and followed for 43.81 ± 1.21 months. The outcome was determined by the combination of cardiac death, heart transplantation, or stroke.

**FINDINGS:**

After the follow-up, 15 patients (22%) presented adverse cardiovascular events. Only left ventricular ejection fraction (LVEF) [heart rate at rest (HR): 0.935, 95% CI 0.878 to 0.994; p = 0.033] and sTNFR2 (HR: 1.002, 95% CI 1.001 to 1.003; p = 0.006) remained as independent predictors of adverse cardiovascular events. The optimal cutoff point to identify these patients was the value of 1784.00 pg/mL. There was a significant difference between the groups with lower and higher sTNFR2 levels (long-rank < 0.001).

**MAIN CONCLUSIONS:**

High serum levels of sTNFR2, together with lower LVEF, are strong independent predictors of adverse cardiovascular events in CCC, making them valuable for risk stratification.

Chagas disease (CD) is an infection caused by the protozoan *Trypanosoma cruzi*,^1^ being considered a neglected tropical disease. Furthermore, the disease is closely linked to poverty and endemic in resource-limited areas, especially in Latin America Currently, CD affects approximately 6 to 7 million people worldwide,^2^ and nowadays, due to the increase in immigration, patients infected with *T. cruzi* also live in Europe, and North America.^3^

Among the manifestations of the disease, chronic Chagas cardiomyopathy (CCC) is the most severe clinical form of the disease, affecting approximately 20-30% of cases, leading to heart failure, complex arrhythmias, thromboembolism, and sudden cardiac death.^4^

One possible marker of disease severity is the response of the host’s immune system to *T. cruzi*, reflecting the balance between the production of inflammatory and anti-inflammatory cytokines.^5^ Thus, an impaired cytokine network has been linked with increased morbidity from the disease.^6^ Patients with CCC have a strong immune response and high concentration of inflammatory cytokines, including the tumour necrosis factor (TNF),^7^ ^,^ ^8^ which contributes to the worse prognosis.

TNF is a pro-inflammatory mediator in immunological processes and acts through its TNF receptor 1 (TNFR1) and TNF receptor 2 (TNFR2),^9^ which in turn may initiate a variety of processes, including cell proliferation, gene activation or cell death.^10^ These receptors may be found in the circulation in a soluble form, being soluble TNF receptor 1 (sTNFR1) and soluble TNF receptor 2 (sTNFR2).^11^ However, according to clinical and experimental studies, the presence of this inflammatory cytokine due to persistent parasitic stimulation contributes to the progression of CD, and may even determine the evolution to CCC.^12^ ^,^ ^13^ Furthermore, the progression of CD is still not fully understood. Given the central relevance of biomarkers in the pathogenesis of CD, the present study was addressed to verify the association between sTNFRs and cardiac function, and to determine the prognostic value of sTNFRs in patients with CCC.

## SUBJECTS AND METHODS

This is a prospective observational study in which patients with CCC were selected and followed up in clinical consultations scheduled at an Outpatient Reference Centre for Chagas disease in November 2013. The research was approved by the Institutional Ethics Committee (CAAE: 03993912.6.0000.5149) and all patients gave their written informed consent before participating in the study. The STrengthening the Reporting of Observational studies in Epidemiology (STROBE) checklist for cohort studies^14^ was used to report the results.

To be included, patients should be diagnosed with CD by, at least, two serological tests with different methodologies. In addition, patients should also have electrocardiographic and/or echocardiographic signs compatible with CCC,^15^ and present a stable clinical condition, defined as no acute exacerbation of the disease in the last three months. Patients undergoing trypanocidal treatment, with other parasitic diseases, co-infections, autoimmune diseases, respiratory or renal diseases, stroke, musculoskeletal limitations, those unable to perform the study evaluation procedures, and those who underwent blood transfusions within the last six months were excluded.

At baseline, the blood sample was collected and the eligible patients underwent echocardiography. The samples were placed in a test tube without additives and left at room temperature for 40 min and then at 4ºC for 30 min. Serum was obtained by centrifugation at 2,000 rpm for 10 min at 4ºC. After collection, serum samples were stored in a freezer at -80ºC. The dosage of sTNFR1 and sTNFR2 was done using serum samples using the Cytometric Bead Array (CBA), and the levels of sTNFR1, sTNFR2 were quantified in serum samples from individuals using the Human Flex (BD™ Cytometric Bead Array Human Soluble TNFRI Flex Set, with a limit of detection of 5.2 pg/mL, and BD™ Cytometric Bead Array Human Soluble TNFRII Flex Set, with a limit of detection of 1.4 pg/mL), following the manufacturer’s instructions. The spheres were acquired within 24 h using the FACScalibur flow cytometer (Becton Dickinson) and analysed using the FCAP Array software (Becton Dickinson).

Echocardiography was performed to evaluate the cardiac function and was guided by the recommendations of the American Society of Echocardiography.^16^ The left ventricular ejection fraction (LVEF), assessed by Simpson’s method, and the end-diastolic volume (LVDD) were the target variables for quantifying the degree of systolic function and left ventricular dilation. Systolic dysfunction was defined by LVEF less than 52 and 54% for men and women, respectively.^16^

*Follow-up period* -The patients were followed up through clinical consultations every three months for four years and in case of non-attendance to the consultation, a telephone call was made where the patients were asked about clinical worsening, hospitalisations, and occurrence of adverse events. The primary outcome was defined as cardiac death, heart transplantation, or stroke.

*Statistical analysis* - Data were analysed using SPSS software, version 20.0 (Chicago, IL). Data distribution was verified using the Kolmogorov-Smirnov test. Descriptive analysis was expressed as mean and standard deviation or median and interquartile range (continuous variable) and absolute number and percentage (categorical variables).

At baseline, the association between sTNFR and LVEF was verified using the Pearson or Spearman correlation tests. The accuracy of the sTNFR in identifying systolic dysfunction in CCC patients was verified by the receiver operating curve (ROC) curve. Differences in the sTNFR values between patients with systolic dysfunction and preserved LVEF were verified by T-test for independent samples or Mann-Whitney. The significance level was set at 5%.

In the follow-up analysis, the predictors of adverse events were verified by Cox uni- and multivariate regression. Univariate analysis was performed with sTNFR and well-established prognostic factors for patients with CCC such as age, sex, NYHA functional class, and echocardiographic parameters.^17^ ^,^ ^18^ Variables associated with adverse events in the univariate analysis (p < 0.1) were included in the multivariate model. A ROC curve was further performed to determine the optimal cut-off points for the variables that remained in the final Cox model to identify patients at risk for adverse cardiovascular events. Optimal cut-off points were obtained using the Youden Index and used in the Kaplan-Meier diagram. The sensitivity, specificity, positive and negative predictive values, and their respective 95% confidence intervals (CI) were obtained using the software MedCalc, version 13.1.2.0 (MedCalc Software, Ostend, Belgium).

## RESULTS

Seventy-nine patients were included in this study. Ten patients were excluded due to comorbidities, such as previous stroke, respiratory diseases, and heart disease of other causes (n = 4), and lack of interest in participating in the study (n = 6). A total of sixty-nine patients were included, with a mean age of 53.7 (± 9.66) years. Most were female, in New York Heart Association (NYHA) functional class I. Demographic, clinical, and echocardiographic characteristics, as well as sTNFRs levels, are depicted in Table I.

**TABLE I:** Baseline characteristics of the sample (n = 69)

Variable	Value
Age (years)	53.70 ± 9.66
Female sex, n (%)	41 (59.4)
NYHA functional class, n (%)	
I	43 (62.3)
II	26 (37.7)
Stage classification, n (%)	
B1	22 (31.9)
B2	26 (37.7)
C	21 (30.4)
Right bundle branch block, n (%)	52 (75.7)
Anterosuperior divisional block, n (%)	40 (58.8)
Diabetes, n (%)	11 (15.9)
Medications, n (%)	
Diuretics	56 (81.1)
ACE inhibitor	54 (78.3)
Antiarrhythmics	32 (46.4)
Angiotensin receptor blockers	28 (40.6)
Beta-blockers	18 (26.1)
Anticoagulants	14 (20.3)
Digitalis	10 (14.5)
Calcium channel blockers	08 (11.6)
BMI (kg/m^2^)	23.94 (21.74 - 26.61)
HR (bpm)	66.51 ± 1.72
SBP (mmHg)	120.00 (110.00 - 140.00)
DBP (mmHg)	80.00 (70.00 - 80.00)
LVEF (%)	47.84 ≥ 16.6
Systolic dysfunction, n (%)	45 (65)
LVDd (mm)	58.01 ± 9.10
E/e’ratio^*^	7.81 (5.96 - 11.55)
sTNFR1 (pg/mL)	367.38 (209.05 - 505.69)
sTNFR2 (pg/mL)	1644.66 (277.27 - 2596.17)

Data presented as mean ± standard deviation, median, and interquartile range or absolute number (percentage). NYHA: New York Heart Association; ACE: angiotensin-converting enzyme; BMI: body mass index; HR: heart rate at rest; SBP: systolic blood pressure; DBP: diastolic blood pressure; LVEF: left ventricular ejection fraction; LVDd: left ventricular end-diastolic diameter; E/e’ ratio: ratio of the early diastolic transmitral flow velocity to early diastolic mitral annular velocity; sTNFR: soluble tumour necrosis factor receptor;^*^ data was collected from only 30 patients.

**Fig. 1:**
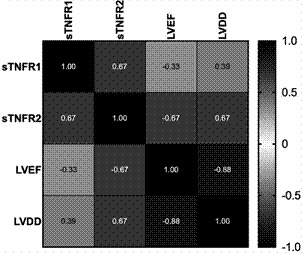
correlation matrix between soluble receptors, left ventricular ejection fraction (LVEF), and left ventricular end-diastolic diameter (LVDD). sTNFR1: soluble TNF receptor 1; sTNFR2: soluble TNF receptor 2.

The sTNFR1 and sTNFR2 correlated with LVEF and LVDD. The correlation analysis is shown in Fig. 1. The concentration of both sTNFR1 and sTNFR2 was higher in patients with systolic dysfunction (p < 0.001 for both). Only sTNFR2, not sTNFR1, significantly correlated with age (r = 0.332, p = 0.002).

After the follow-up period (43.81 ± 1.21 months), 15 patients (22%) had adverse cardiovascular events. Nine patients died, four patients had a stroke, and two patients had heart transplants. There were no missing data. Patients with adverse cardiovascular events had higher levels of sTNFR2, lower LVEF, higher LVDd, and a higher E/e’ ratio when compared to those without adverse cardiovascular events. Differences between groups are detailed in Table II.

**TABLE II:** Demographic, clinical, echocardiographic, and functional characteristics of the patients with and without adverse cardiovascular events

Variable	Without adverse event (n = 54)	With adverse event (n = 15)	p-value
Age (years)	55.09 ± 9.64	48.67 ± 8.20	0.022
Female sex, n (%)	35 (85.4)	06 (14.6)	0.077
NYHA class, n (%)			
I	33 (76.5)	10 (23.5)	0.469
II	21 (80.8)	05 (19.2)	
BMI (kg/m^2^)	24.41 (22.00 - 28.57)	24.61 (22.77 - 30.42)	0.590
HR (bpm)	69.83 ± 18.05	66.33 ± 10.99	0.478
SBP (mmHg)	125.00 (110.00 - 140.00)	120.00 (100.00 - 120.00)	0.055
DBP (mmHg)	80.00 (70.00 - 80.00)	80.00 (70.00 - 80.00)	0.066
LVEF (%)	51.73 ± 16.69	35.13 ± 7.55	<0.001
LVDd (mm)	55.99 ± 9.04	64.60 ± 5.57	0.001
E/e’ratio^*^	7.60 (6.56 - 11.67)	10.32 (4.81 - 11.51)	0.915
sTNFR1 (pg/mL)	335.47 (187.68 - 497.93)	404.13 (290.04 - 608.90)	0.065
sTNFR2 (pg/mL)	359.29 (253.26 - 2237.20)	2668.23 (2060.55 - 3687.95)	<0.001

Data presented as mean ± standard deviation, median, and interquartile range or absolute number (percentage). Values highlighted in bold were statistically differences between groups (p < 0.05). NYHA: New York Heart Association; ACE: angiotensin-converting enzyme; BMI: body mass index; HR: heart rate at rest; SBP: systolic blood pressure; DBP: diastolic blood pressure; LVEF: left ventricular ejection fraction; LVDd: left ventricular end-diastolic diameter; E/e’ ratio: ratio of the early diastolic transmitral flow velocity to early diastolic mitral annular velocity; sTNFR: soluble tumour necrosis factor receptor; ^*^ data was collected from only 30 patients.

In the univariate Cox analysis, sTNFR1, sTNFR2, age, male sex, LVEF, and LVDD are associated with a worse prognosis (Table III). In the final multivariate model, only LVEF [heart rate at rest (HR): 0.935, 95% CI 0.878 to 0.994; p = 0.033] and sTNFR2 (HR: 1.001, 95% CI 1.001 to 1.001; p = 0.006) remained as independent predictors of adverse cardiovascular events.

**TABLE III:** Factors associated with worse prognosis in the univariate Cox regression

Variable	Univariate analysis		
	HR	95% CI	p-value
Age	0.943	0.895 - 0.994	0.030
Male sex	0.393	0.140 - 1.104	0.076
NYHA	0.802	0.274 - 2.347	0.688
BMI	1.020	0.911 - 1.142	0.727
LVEF	0.930	0.890 - 0.972	0.001
LVDd	1.119	1.041 - 1.204	0.002
E/e’ ratio	1.008	0.887 - 1.145	0.907
sTNFR1	1.002	1.000 - 1.004	0.016
sTNFR2	1.001	1.000 - 1.001	0.001

Values highlighted were included in the Cox multivariate model. NYHA: New York Heart Association; BMI: body mass index; LVEF: left ventricular ejection fraction; LVDd: left ventricular end-diastolic diameter; E/e’ ratio: ratio of the early diastolic transmitral flow velocity to early diastolic mitral annular velocity; sTNFR: soluble tumour necrosis factor receptor; HR: heart rate at rest; CI: confidence interval.

**Fig. 2:**
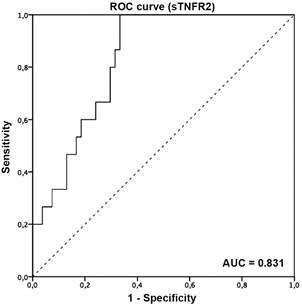
accuracy of sTNFR2 in predicting adverse cardiovascular events in patients with chronic Chagas cardiomyopathy (CCC).

The area under the curve (AUC) for sTNFR2 levels to identify patients with adverse cardiovascular events was 0.831 (95% CI: 0.73 to 0.92) (Fig. 2), and the optimal cut-off point to identify these patients was 1784.00 pg/mL (sensitivity and specificity of 100% and 67%, respectively). The cut-off, sensitivity, specificity, positive predictive values (PPV), and negative predictive values (NPV) for sTNFR2 are shown in Table IV.

**TABLE IV:** Cut-off points, the area under the receiver operating characteristic (ROC) curve, sensitivity, specificity, and positive and negative predictive values of sTNFR2 in identifying patients with cardiovascular adverse events

	AUC (95% CI)	Cut-off point	Sensitivity (95% CI)	Specificity (95% CI)	NPV (95% CI)	PPV (95% CI)
sTNFR2	0.83 (0.73-0.93)	1784.00 pg/mL	100% (78-100%)	67% (52-78%)	100% (90-100%)	45% (28-63%)

AUC: area under the ROC curve; CI: confidence interval; NPV: negative predictive value; PPV: positive predictive value; sTNFR: soluble tumour necrosis factor receptor.

**Fig. 3:**
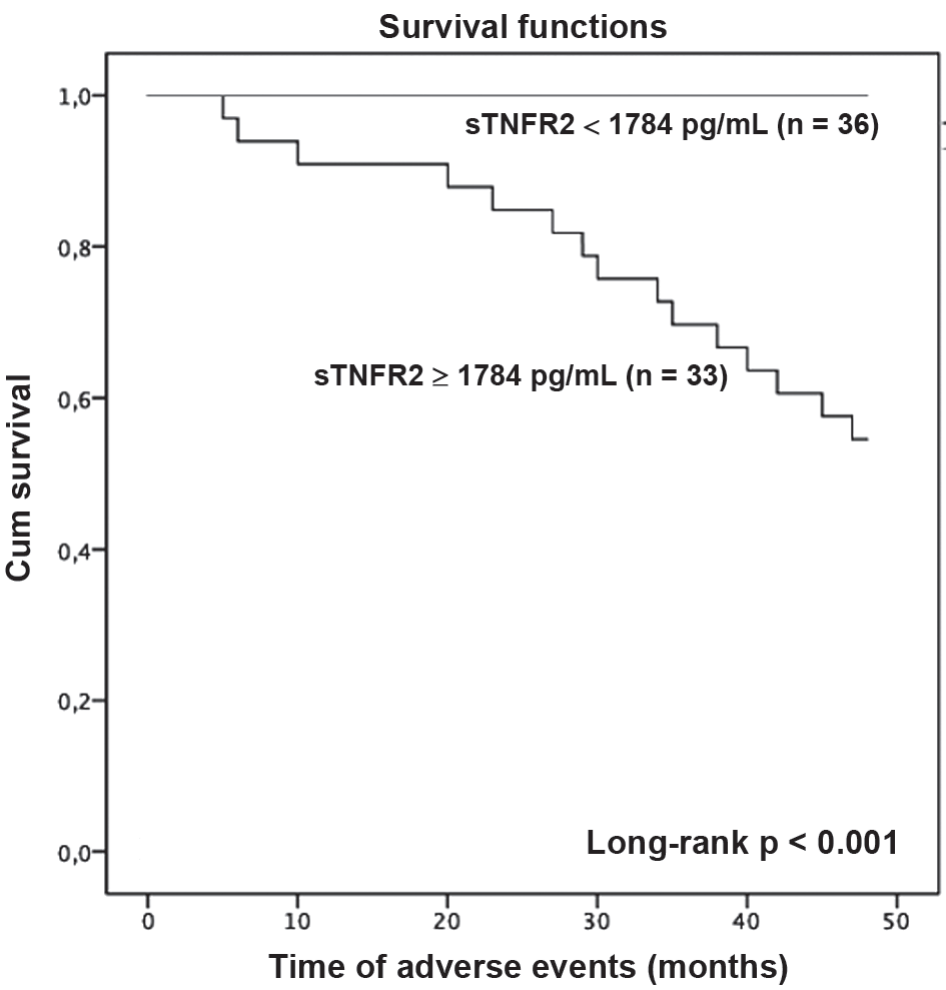
the Kaplan-Meier diagram compares groups with lower (< 1784.00) and higher (> 1784.00) levels of sTNFR2. The cut-off points were determined using the receiver operating characteristic (ROC) curve.

On the Kaplan-Meier diagram, there was a significant difference between groups with lower (< 1784 pg/mL; n = 36 patients) and higher (≥ 1784 pg/mL; n = 33 patients) levels of sTNFR2 (long-rank < 0.001) (Fig. 3).

## DISCUSSION

There is a growing interest in studies involving blood biomarkers in patients with CD to assess therapeutic responses and prognosis of the disease. The TNF/TNFR1 pathway plays an important role in controlling parasitaemia and inducing inflammation, while its soluble receptors (sTNFR1 and sTNFR2) play an important role in neutralising this cytokine.^19^ ^,^ ^20^ In the present study, we found that (1) sTNFR2, not sTNFR1, and LVEF are independent predictors of adverse cardiovascular events, (2) the optimal cut-off point for sTNFR2 levels to identify patients at risk of adverse cardiovascular events was 1784 pg/mL and (3) patients with serum levels of sTNFR2 below 1784 pg/mL 100% of chance of not having adverse cardiovascular events.

The detection of sTNFRs is a useful tool to gain insight into immune processes and provide valuable information about a variety of pathological conditions, such as infections, autoimmune disorders and cancer.^21^ Those soluble receptors are increased in other cardiopathies. In patients with coronary artery disease,^22^ sTNRF1 and sTNF2 correlated with B-type natriuretic peptide (BNP), a marker of cardiac overload.

Identifying the levels of these receptors is already a reality in assessing the prognosis of many clinical conditions, such as heart failure,^23^ and myocardial infarction.^24^ In a multicentre study with 1,200 patients with heart failure, one study^23^ demonstrated that both sTNFR1 and sTNFR2 levels were different between survivors and non-survivors (p < 0.001 for both). In the final Cox multivariate analysis with a variety of cytokines and receptors, sTNFR2 (Relative risk = 3.53; 95% CI = 2.08 to 6.00; p < 0.001), NYHA class (Relative risk = 2.38; 95% CI = 1.26 to 4.50; p = 0.007), and LVEF (Relative risk = 0.94; 95% CI = 0.90 to 0.98; p = 0.004) remained a significant predictor of survival. Similarly, the present study found that sTNFR2 and LVEF were independent predictors of poor outcomes. However, the NYHA functional class did not predict adverse events in our study. We hypothesised that the functional class of the patients included in our study was slightly impaired, as is typically observed in patients with CCC.

Another study^24^ reported that both sTNFR1 and sTNFR2 were increased after myocardial infarction and were predictors of an adverse clinical event within 24 months (log-rank < 0.001 for both). These findings suggest that increases in those soluble receptors are linked to myocardial damage, identified by the weak but significant association between peak sTNFR1 and peak sTNFR2 with LVEF, infarct size, and left ventricular end-diastolic volume (p < 0.05 for all). Our study also found similar results, demonstrating a significant correlation between biomarker levels and cardiac function.

In patients with CD, Torres et al.^25^ reported increased levels of sTNFR1 and sTNFR2, especially in the chronic phase of the disease, when compared to non-infected individuals. In our study, both soluble receptors correlated with LVEF and LVDD at baseline, demonstrating the association between soluble receptors and cardiac function. However, only sTNFR2 correlated with age. The correlation between age and soluble receptors is expected due to an inflammatory phenotype typical of aging featuring increases in inflammatory markers, suggesting the induction of chronic low-level inflammation. Deswal et al.^23^ demonstrated a significant correlation between receptors and age in a multicentre study. In contrast, a recent study^26^ found no correlation between age and sTNFR1 and a weak correlation between age and sTNFR2 (r = 0.11, p < 0.05) in healthy subjects (n = 413). As in our study, the patients were relatively young (43 ± 12 years), most of them non-elderly, and this may have influenced the results. We believe that the significant correlation between soluble receptors and age is more pronounced in elderly patients.

However, no previous study has analysed the prognostic properties of sTNFRs in patients with CCC, and the present study showed sTNFR2 as an independent predictor of adverse cardiovascular events in those patients. A study carried out by Silva et al.^27^ demonstrates that higher plasma levels of sTNFR1 and sTNFR2 were associated with a worse systolic function (R^2^ = 0.10; p = 0.008 and R^2^ = 0.44; p < 0.001) and cardiac dilation (R^2^ = 0.13; p = 0.002 and R^2^ = 0.43; p < 0.001). We hypothesised that the presence of the *T. cruzi* protozoan induces the production of the cytokine TNF, and its presence is positively associated with myocardial damage and fibrosis, characterising the loss of contractile cells and causing tissue damage.^28^

Furthermore, our study also shows that male sex, advanced age, and LVEF were associated with poor prognosis. It has been reported^29^ for more than two decades that male patients with CCC have a worse prognosis, probably due to the higher prevalence of myocardial dysfunction. Regarding age, with advances in treatment, greater knowledge about the disease, and population aging, people with CCC are living longer. It is expected that people with advanced age tend to have a higher probability of death and adverse outcomes.

However, in the final model, only sTNFR2 and LVEF remained as independent predictors. The LVEF and, consequently, systolic dysfunction are well-established predictors of mortality.^30^ The loss of cardiac function associated with dilation of the cardiac chambers leads to the worst outcome. As a main finding, even when adjusted for LVEF, the sTNFR2 remained a predictor of morbidity in the population.

Moreover, our study first reported a sTNFR2 cut-off point to predict adverse cardiovascular events in patients with CCC. Due to its high negative predictive value, patients who have serum levels of sTNFR2 below 1784 pg/mL have a 100% chance of not having a short-term adverse cardiovascular event. In this scenario, the sTNFR2 can be used to assist in risk stratification and patient screening. In contrast, values of sTNFR2 above the cut-off have low positive predictive value (45%) and patients with this value should be further investigated.

The present study had some limitations. Firstly, the sample was small. In addition, most of them had preserved functional, however, it is a common feature in patients with CD, since they usually come from regions with low Human Development Index and work with activities that demand physical effort. Patients with diabetes were also included in the present study. Although diabetes can increase soluble receptor levels, we included these patients because they are representative of the CCC population. As a strength, the present study contributes to the prognostic evaluation of patients with CCC, generating cut-off points that help health professionals stratify the risk of these patients developing adverse cardiovascular events. The prognosis of CCC is difficult to establish due to wide inter-individual variability, and the identification of valuable biomarkers could aid in the achievement of a better outcome. Due to the growing interest in the role of biomarkers in CCC, scoping or systematic reviews to identify knowledge gaps or summarise the main findings are necessary.

*In conclusion* - There is an association between both sTNFR and systolic function. The high serum level of sTNFR2, together with the systolic function, was an independent predictor of adverse cardiovascular events in CCC. The sTNFR2 can help understand the pathophysiology of CCC and may be used in the risk stratification of these patients.
